# The *Helicobacter pylori dupA*: A Novel Biomarker for Digestive Diseases

**DOI:** 10.3389/fmed.2014.00013

**Published:** 2014-05-28

**Authors:** Amin Talebi Bezmin Abadi

**Affiliations:** ^1^Department of Medical Microbiology, University Medical Center Utrecht, Utrecht, Netherlands

**Keywords:** biomarker, *Helicobacter pylori*, *dupA*, digestive system, gene cluster

## Introduction

*Helicobacter pylori* (*H. pylori*) infection affects approximately half of the world’s population. Unless eradicated, it remains in the stomach throughout life. After 1983, discovery of *H. pylori* as persistent resident of gastric mucosa, changed the traditional thoughts and perspectives regarding (i) having sterile stomach lifelong, (ii) infectious potential links existence with various extra gastric disorders, and (iii) ongoing researches on *H. pylori*-induced diseases (1). Undeniably, colonization with pathogenic *H. pylori* results in severe gastroduodenal disorders. *H. pylori* is well-recognized as an established causative agent associated with a wide variety of upper gastroduodenal diseases ranged from a chronic gastritis to gastric cancer. Surprisingly, gastric colonization with *H. pylori* induces superficial gastritis in all infected individuals, while only a minority develops to severe symptomatic diseases (2). Notably, rationale underlying this unique distribution of diseases is driven by a sophisticated and mysterious interplay between *H. pylori* and its host. Broadly defined, the certain pattern of *H. pylori*-induced digestive disease is strongly influenced by bacterial virulence factors which draw the final outcome of infection (2, 3). It is now firmly established that biomarkers allow us for having an early diagnosis and prediction of medical conditions. Moreover, biomarkers provide opportunity to modify current available protocols to manage the infection and its associated outcomes (4). In this case, a biologic biomarker can be used for early diagnosis of certain digestive disease (e.g., gastric cancer, duodenal ulcer); and even identification of high-risk population for disease prevention. As such, biomarkers are becoming increasingly important tools in clinical settings. Thus, potential applications of biomarkers in infectious diseases such as predicting outcomes would be an interesting area of ongoing research. With this regard, the search for relevant biomarkers that diagnose/predict a clinical condition among the *H. pylori*-infected patients is a challenging area of research. Following several suggested *H. pylori* biomarkers for certain digestive diseases over the past years (5–8), still a biomarker capable of predicting definitive digestive diseases outcome is lacking for clinical settings.

## Definition and Application of the Opinion

**Figure 1:**
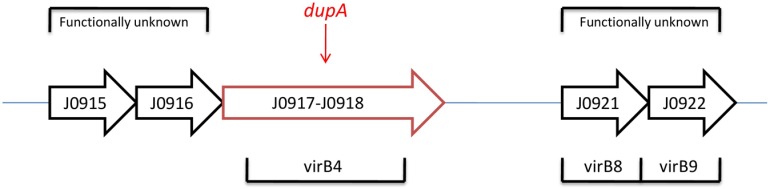
Full type IV secretion system (T4SS) in the *H. pylori* plasticity zone in J99*.

In this new opinion, under condition of identification of complete duodenal ulcer promoting (*dupA*) gene, our knowledge about biomarker application in *H. pylori*-induced diseases such as duodenal ulcer and gastric cancer will be influenced greatly. Accordingly, we propose a logical and practical opinion that detection of full length (*dupA* and its flanking gene) (Figure 1) *H. pylori dupA* gene by simple multiplex PCR assay can be used to predict digestive diseases outcome and solve the above-mentioned problems. To examine *H. pylori dupA* as biomarker that can serve as an indicator of the digestive disease potential, our opinion can be easily be evaluated in a cohort of population with different disease groups including gastric cancer, duodenal ulcer, and gastritis. Furthermore, primer set which can pick whole *dupA* gene in addition to both left and right sides of the *dupA* gene (Figure 1) would be preferable to investigate likely involvement of this cluster in determining final diseases outcome. To date, an actual function of *dupA* is still not fully understood. Moreover, flanking genes to *dupA* (virB4, virB8, and virB9) are also not determined. Strikingly, the opinion stands the best situation of being confirmed if *dupA* as part of (Type 4 secretion system) T4SS can show *in vivo* activity.

## Discussion

Current opinion would be confirmed by a finding that *dupA* is forming T4SS in combination of those flanking genes. Indeed, function of flanking genes to *dupA* (virB4, 8, and 9) are not determined yet (Figure 1). Hence, this interesting gap promotes researchers to examine it within a cohort study. Of note, *dupA* gene encodes homologs of virB4 ATPase which current knowledge found it involved in DNA transfer/uptake. Involvement of T4SS in pathogenesis of *H. pylori* is an apparent fact. Accordingly, current evidences are indicating on potential role of *dupA* to form a functional T4SS. One further step would be to check those flanking genes in combination of *dupA*; an opinion which might be able to answer those unclear points regarding *dupA* after its introduction in 2005 to now (9, 10). Notably, *dupA* and its flanking genes are located in plasticity region (PR) of the *H. pylori* genome (11). It is important to point out that the *vir* genes exist before and after the region of the *dupA* locus and the surrounding six *vir* gene homologs (*virB8, virB9, virB10, virB11, virD4*, and *virD2*) are important in forming a novel putative T4SS (*tfs3a*). As a result, *H. pylori* strains containing full *dupA* are bound to be virulent due to the ability of building a complete T4SS. In a continuous cluster in *H. pylori* strain J99, the PR has been reported to range from *jhp0914* to *jhp0961* (Figure 1) (12). In *H. pylori* genome, PR is an area where G + C content is lower than that of the rest (35% compared with 39%), indicating on variable genes which mostly are virulence associated (11). Thus, the polymorphism pattern in this area of PR can be considered as related with different set of bacterial virulence. That would be rationally possible that sequential contribution of these genes be involved to determine final outcome of the *H. pylori* in colonized individuals. However, this article is first to present this possibility; an opinion can be promising if proven after those mentioned in *in vivo* tests.

## Conclusion

This paper is an invitation for having a different look in to the biomarkers for digestive diseases. Current opinion encourage using *H. pylori dupA* as a predicting tool to screen certain types of digestive diseases such as duodenal ulcer and gastric cancer.

## Conflict of Interest Statement

The author declares that the research was conducted in the absence of any commercial or financial relationships that could be construed as a potential conflict of interest.
